# Augmented reality navigation in spine surgery: a systematic review

**DOI:** 10.1007/s00701-021-04708-3

**Published:** 2021-01-28

**Authors:** Gustav Burström, Oscar Persson, Erik Edström, Adrian Elmi-Terander

**Affiliations:** 1 Department of Clinical Neuroscience, Karolinska Institutet, Stockholm, Sweden; 2 Department of Neurosurgery, Karolinska University Hospital, 171 76 Stockholm, Sweden

**Keywords:** Accuracy, Augmented reality, Pedicle screw, Systematic review, Surgical navigation, Virtual path tracking

## Abstract

**Background:**

Conventional spinal navigation solutions have been criticized for having a negative impact on time in the operating room and workflow. AR navigation could potentially alleviate some of these concerns while retaining the benefits of navigated spine surgery. The objective of this study is to summarize the current evidence for using augmented reality (AR) navigation in spine surgery.

**Methods:**

We performed a systematic review to explore the current evidence for using AR navigation in spine surgery. PubMed and Web of Science were searched from database inception to November 27, 2020, for data on the AR navigation solutions; the reported efficacy of the systems; and their impact on workflow, radiation, and cost-benefit relationships.

**Results:**

In this systematic review, 28 studies were included in the final analysis. The main findings were superior workflow and non-inferior accuracy when comparing AR to free-hand (FH) or conventional surgical navigation techniques. A limited number of studies indicated decreased use of radiation. There were no studies reporting mortality, morbidity, or cost-benefit relationships.

**Conclusions:**

AR provides a meaningful addition to FH surgery and traditional navigation methods for spine surgery. However, the current evidence base is limited and prospective studies on clinical outcomes and cost-benefit relationships are needed.

## Introduction

Compared to conventional free-hand (FH) surgical techniques, computer-assisted surgery (CAS) has been shown to improve pedicle screw placement accuracy and is gaining use in spine surgery [41]. Despite the known advantages of CAS, implementation in spine surgery has been slower than corresponding cranial applications, partly due to the relatively complicated and time-consuming setup in spine. A negative impact on OR time and workflow is the most common concern among spine surgeons regarding CAS systems [25]. In conventional navigation, the surgeon must look away from the surgical field into a dedicated navigation screen to verify anatomical positions and landmarks on a virtual 3D representation.

An ideal CAS solution allows for the visualization of the anatomy without obscuring the surgical field or distracting the surgeon. In augmented reality (AR) navigation solutions, the real environment and virtual information is presented in the same field of view [3]. The AR view is an augmentation of reality with virtual content to improve surgical workflow and promote increased adoption of navigation technologies in spine surgery [27].

Current research on AR navigation is at an early stage [28]. One of the first published clinical papers on AR navigation in spine surgery was authored by Wu et al., and explored the use of a projector to display the underlying spine anatomy on the surface of the patient [47]. The system was qualitatively reviewed by surgeons in three patients but further studies on this system have not been published. The first published work on an AR application later brought into clinical trials was performed by Elmi-Terander et al. [22]. This system relied on an AR-enhanced video feed of the surgical field, shown on a monitor. Several preclinical and clinical trials using the same AR approach have followed [2, 8, 16, 17, 19–21, 38]. Later studies have focused on AR systems presented on head-mounted displays instead of monitors, providing AR directly in the surgeon’s field of view [31, 42, 46]. However, the potential benefits of using AR in spine surgery in terms of accuracy, radiation doses, workflow, and cost-benefit have yet to be determined. Previous systematic reviews have partially included spine surgery while focusing on AR surgery in general; however, a focused systematic review of AR navigation for spine surgery is lacking [27, 34, 43].

This study aims to present a systematic review of the current state of AR navigation in spine surgery. We describe the currently available AR navigation interfaces and patient tracking solutions, and summarize the reported accuracies and impact on clinical outcomes, as well as the impact on workflow, radiation, and cost-benefit relationships. Focusing on clinically relevant publications, only studies on patients, cadavers, or cadaveric vertebral models are included.

## Methods

### Search methods and selection process

A systematic search was performed in two databases, “PubMed” and “Web of Science.” All studies written in English from database inception until 27th of November 2020 were included. A combination of the words and phrases (augmented reality), and ((spine or spinal) surgery) or (pedicle screw), was used. Each included study was screened for additional cited and relevant studies to be included in the systematic review. Based on title and abstract, an eligibility screening was performed by two authors. Next, a selection of eligible studies was performed by analyzing full texts. The review followed the Preferred Reporting Items on Systematic Reviews and Meta-analysis (PRISMA) guidelines [29].

### Eligibility criteria and selection process

Inclusion criteria were (1) studies in English; (2) minimum evidence level V using Oxford Centre for Evidence-Based Medicine 2011 Levels of Evidence; (3) AR was used in spine surgery on patients, cadavers, or cadaveric vertebral models; and (4) surgical outcome was reported. Exclusion criteria were (1) review articles or meeting abstracts; (2) articles lacking an available full text; (3) AR used for surgery other than spinal, or solely for biopsies or non-surgical injections; (4) proof-of-concept studies or case-reports reporting single attempts; and (5) studies reporting only qualitative feedback from surgeons.

### Data extraction and quality assessment

Data extraction consisted of author and year of study, type of study subjects, AR interface type, patient tracking solution, and the main findings. Main findings were further sub-divided into accuracy assessment, radiation dose, and cost-benefit analyses. The quality of the available evidence was graded according to the GRADE criteria [24].

## Results

**Fig. 1:**
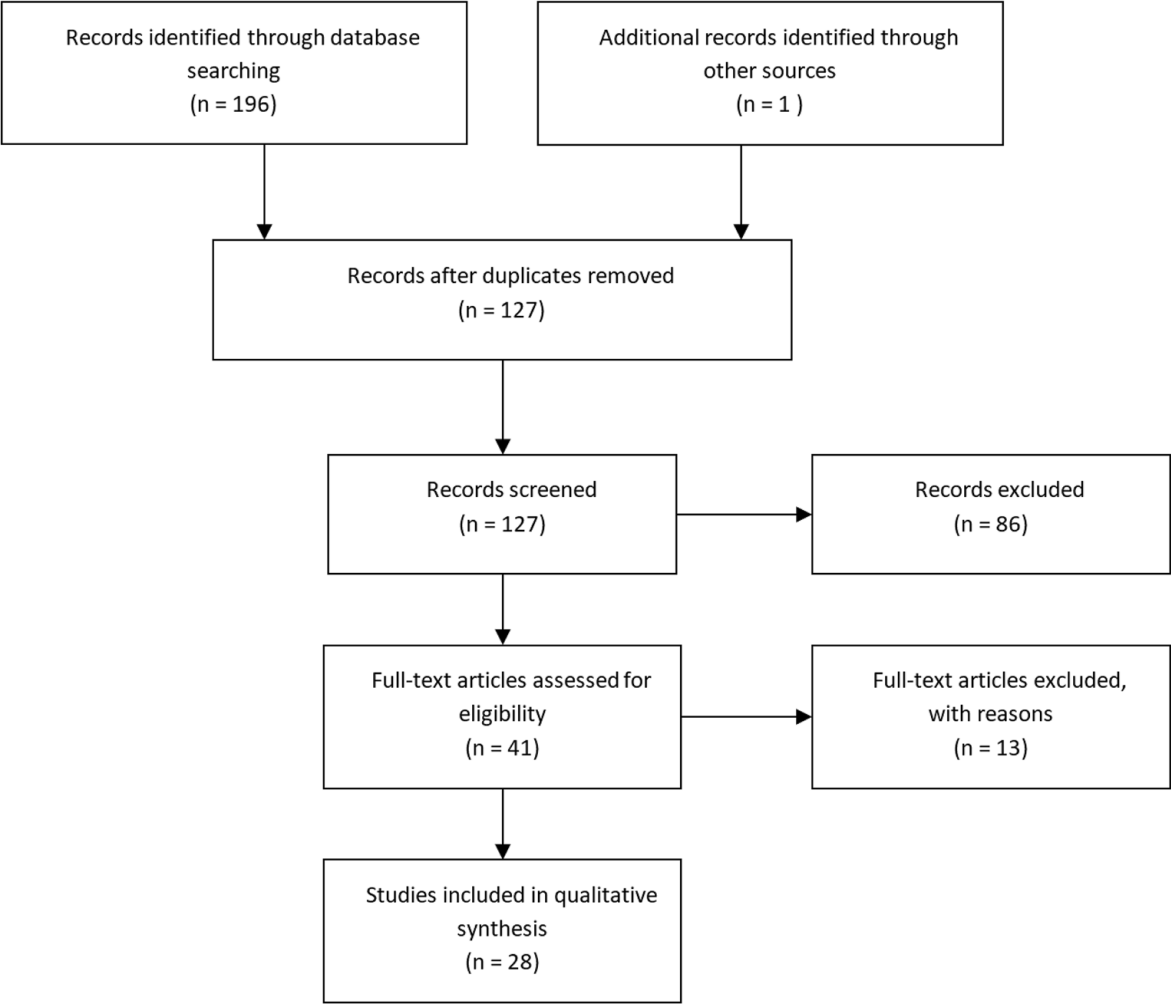
Flowchart of the systematic research in “PubMed” and “Web of Science”

A total of 28 studies were included, as shown in Fig. 1 and detailed in Table 1. The PubMed and Web of science searches returned 100 and 96 results, respectively. After removal of duplicates, 127 studies remained. Based on title and abstracts, 41 studies were selected for inclusion, and full-text versions were obtained. Thirteen studies were excluded from the final analysis since they did not meet the inclusion criteria or met exclusion criteria. After carefully reviewing the bibliography of each of the papers, one additional citation was included. The majority of studies (19 out of 28) concerned pedicle screw placement or pedicle cannulation in thoracolumbar levels (Table 1).

**Table 1:** Studies on augmented reality navigation in spine surgery

Authors	Year	Study type	Study subjects	Procedure	Spine segments	Interface type	Patient registration/tracking type
Abe et al. [1]	2013	Cohort	Phantom + patients	Vertebroplasty	Thoracolumbar	HMD	Preop CT and fluoroscopy/DRF
Elmi-Terander et al. [22]	2016	Controlled	Cadaver	Pedicle screw	Thoracolumbar	Monitor	Intraop CBCT/skin markers
Ma et al. [33]	2017	Cohort	Phantom	Pedicle screw	Lumbar	Projector	Ultrasound/DRF
Elmi-Terander et al. [21]	2018	Cohort	Cadaver	Pedicle screw	Thoracolumbar	Monitor	Intraop CBCT/skin markers
Auloge et al. [2]	2019	RCT	Patients	Vertebroplasty	Thoracolumbar	Monitor	Intraop CBCT/skin markers
Burström et al. [8]	2019	Cohort	Cadaver (pig)	Pedicle screw	Thoracolumbar	Monitor	Intraop CBCT/skin markers
Carl et al. [10]	2019	Cohort	Patients	Multiple	All segments	Microscope	Intraop CT/DRF
Carl et al. [11]	2019	Cohort	Patients	Tumor	Cervical, thoracic	Microscope	Intraop CT/DRF
Carl et al. [13]	2019	Cohort	Patients	Tumor	All segments	Microscope	Intraop CT/DRF
Elmi-Terander et al. [20]	2019	Cohort	Patients	Pedicle screw	Thoracolumbar	Monitor	Intraop CBCT/skin markers
Gibby et al. [23]	2019	Cohort	Phantom	Pedicle screw	Lumbar	HMD (HoloLens)	Manual adjustments/surface tracking
Liebmann et al. [30]	2019	Cohort	Phantom	Pedicle screw	Lumbar	HMD (HoloLens)	Pointer/surface tracking
Liu et al. [31]	2019	Controlled	Phantom	Pedicle screw	Lumbar	HMD (HoloLens)	Two groups: CT or manual/surface tracking
Molina et al. [36]	2019	Cohort	Cadaver	Pedicle screw	Thoracolumbar	HMD	Intraop CT/DRF
Urakov et al. [42]	2019	Controlled	Cadaver	Pedicle screw	Thoracolumbar	HMD (HoloLens)	Manual adjustments/surface tracking
Wanivenhaus et al. [45]	2019	Controlled	Phantom	Rod bending	Lumbosacral	HMD (HoloLens)	Pointer/surface tracking
Wei et al. [46]	2019	RCT	Patients	Kyphoplasty	Thoracolumbar	HMD (HoloLens)	Manual adjustments/surface tracking
Edstrom et al. [17]	2020	Cohort	Patients	Pedicle screw	Thoracolumbar	Monitor	Intraop CBCT/skin markers
Edstrom et al. [16]	2020	Cohort	Patients	Pedicle screw	Thoracolumbar	Monitor	Intraop CBCT/skin markers
Elmi-Terander et al. [19]	2020	Controlled	Patients	Pedicle screw	Thoracolumbar	Monitor	Intraop CBCT/skin markers
Muller et al. [37]	2020	Controlled	Phantom/Cadaver	Pedicle screw	Lumbar	HMD (HoloLens)	Fluoroscopy/DRF
Edström et al. [18]	2020	Controlled	Patients	Pedicle screw	Thoracolumbar	Monitor	Intraop CBCT/skin markers
Peh et al. [38]	2020	Controlled	Cadaver	Pedicle screw	Thoracolumbar	Monitor	Intraop CBCT/skin markers
Carl et al. [12]	2020	Cohort	Patients	Multiple	All segments	Microscope	Intraop CT/DRF
Dennler et al. [14]	2020	Controlled	Phantom	Pedicle screw	Lumbar	HMD (HoloLens)	Manual adjustments/surface tracking
Molina et al. [35]	2020	Cohort	Cadaver	Pedicle screw	Thoracic and lumbosacral	HMD	Intraop CT/DRF
Siemionow et al. [40]	2020	Cohort	Cadaver	Pedicle screw	Thoracolumbar	Projector	Intraop CT/DRF
von Atzigen et al. [44]	2020	Cohort	Phantom	Rod bending	Lumbosacral	HMD (HoloLens)	Surface tracking

*CT* computed tomography, *DRF* dynamic reference frame, *HMD* head-mounted display, *Preop* preoperative, *RCT* randomized controlled trial

### Interfaces

**Fig. 2:**
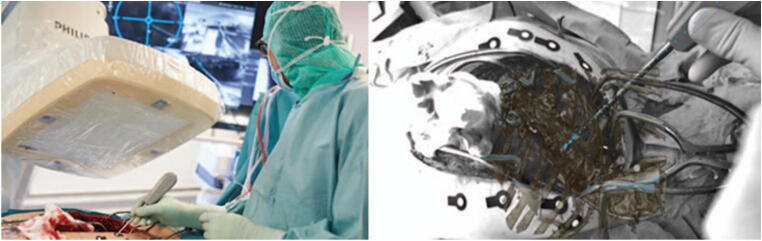
Example of a monitor-based augmented reality (AR) system. To the right, the operating room setup during surgery is seen. To the right, a depiction of the AR interface as seen by the surgeon. The figure is an unmodified reproduction of the work of Elmi-Terander et al., published in the *Spine* [20]

**Fig. 3:**
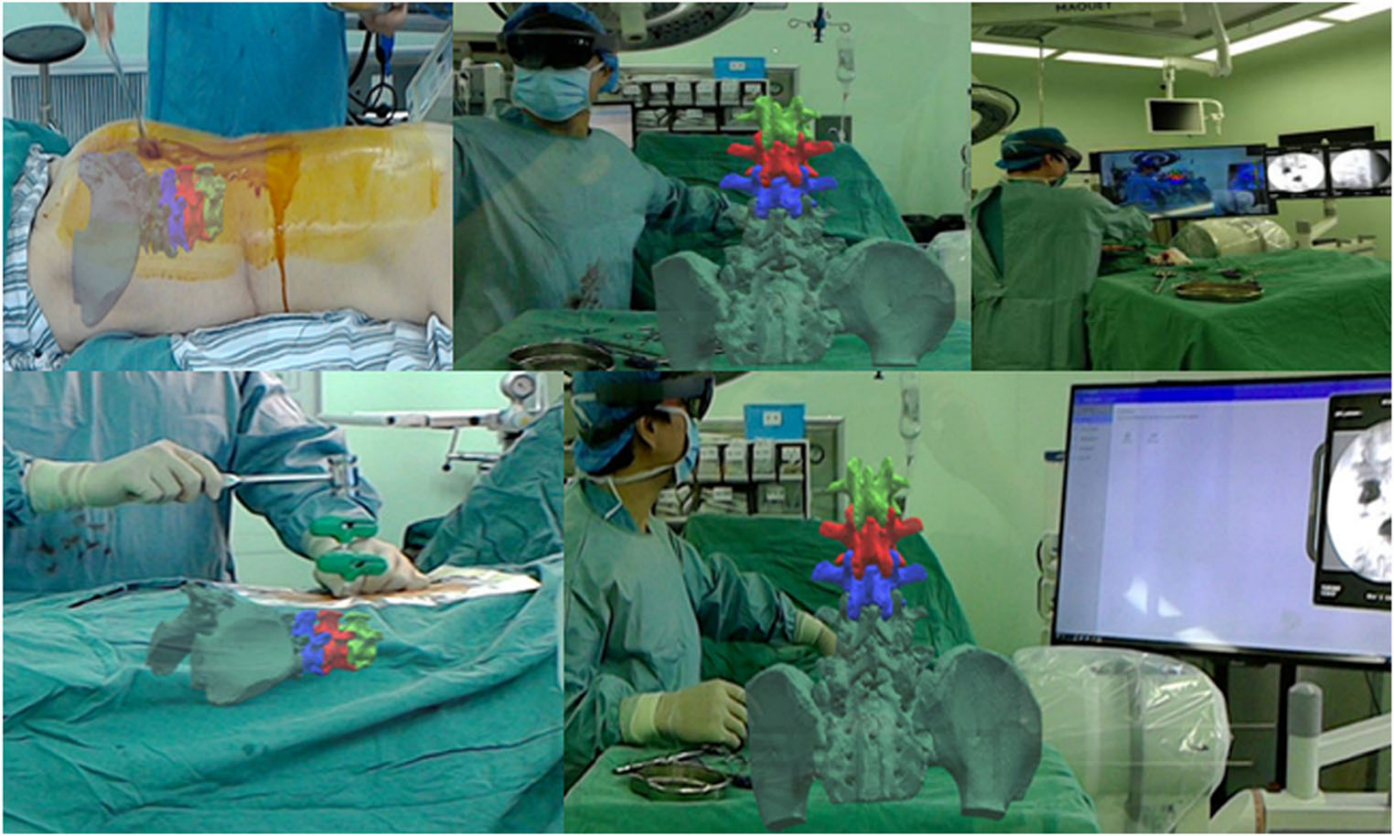
Example of an augmented reality (AR) system using a head-mounted display. Multiple intraoperative views are depicted, with the AR overlay superimposed as viewed by the surgeon. The figure is an unmodified reproduction of the work of Wei et al., published in the *Journal of Orthopaedic Surgery and Research* [46]. It is reproduced under the Creative Commons Attribution License (http://creativecommons.org/licenses/by/4.0/)

**Fig. 4:**
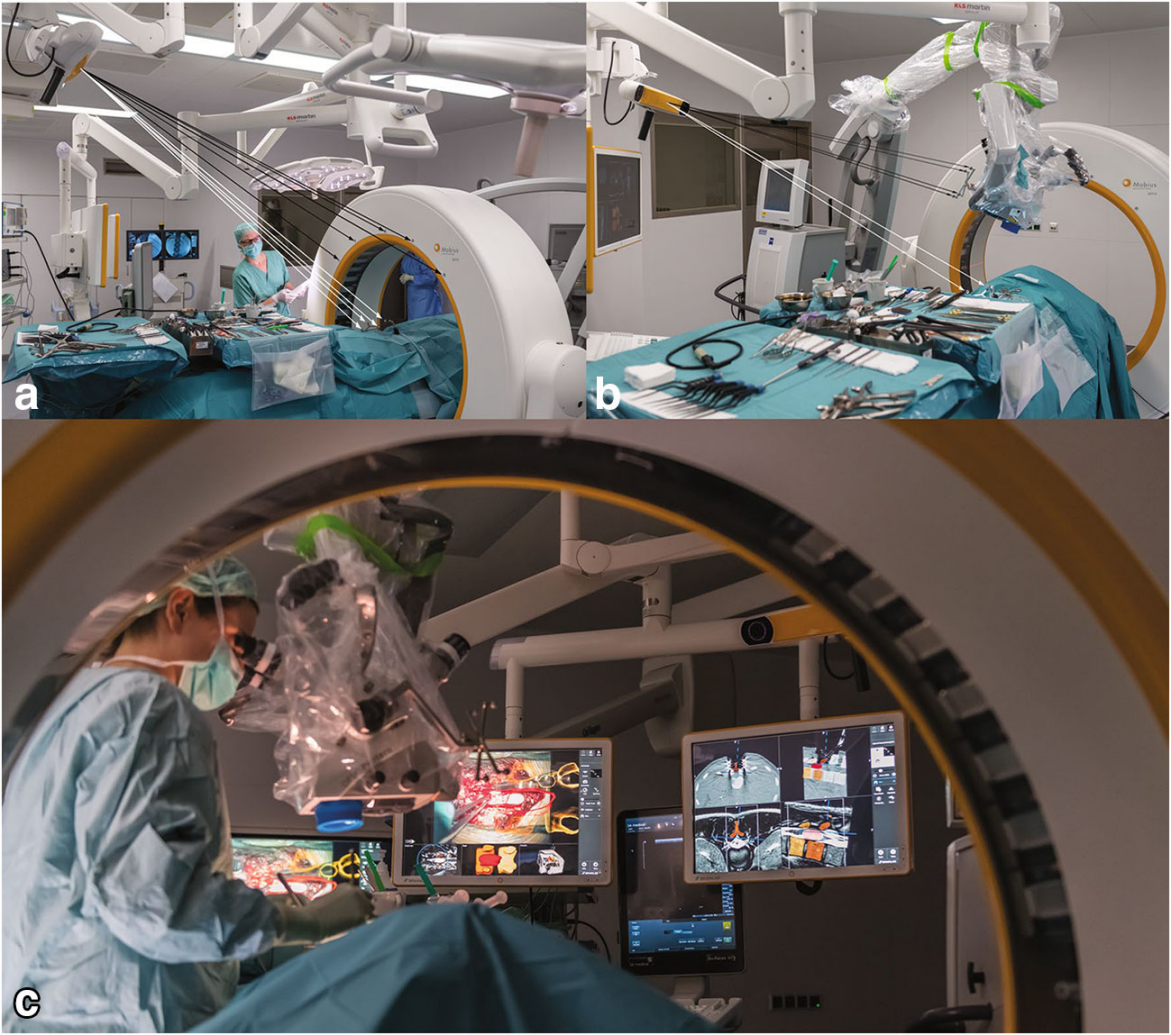
Example of a microscope-based augmented reality system. **a** The operating room setup during patient registration using an intraoperative CT (black arrows) and a dynamic reference frame (white arrows). **b** The operating room setup is seen during surgery with continuous patient tracking (white arrows) and microscope tracking (black arrows). **c** Active surgery is seen with augmented reality views on monitors in the background, mirroring what is seen in the microscope. The figure is an unmodified reproduction of the work of Carl et al., published in the *European Spine Journal* [13]

**Fig. 5:**
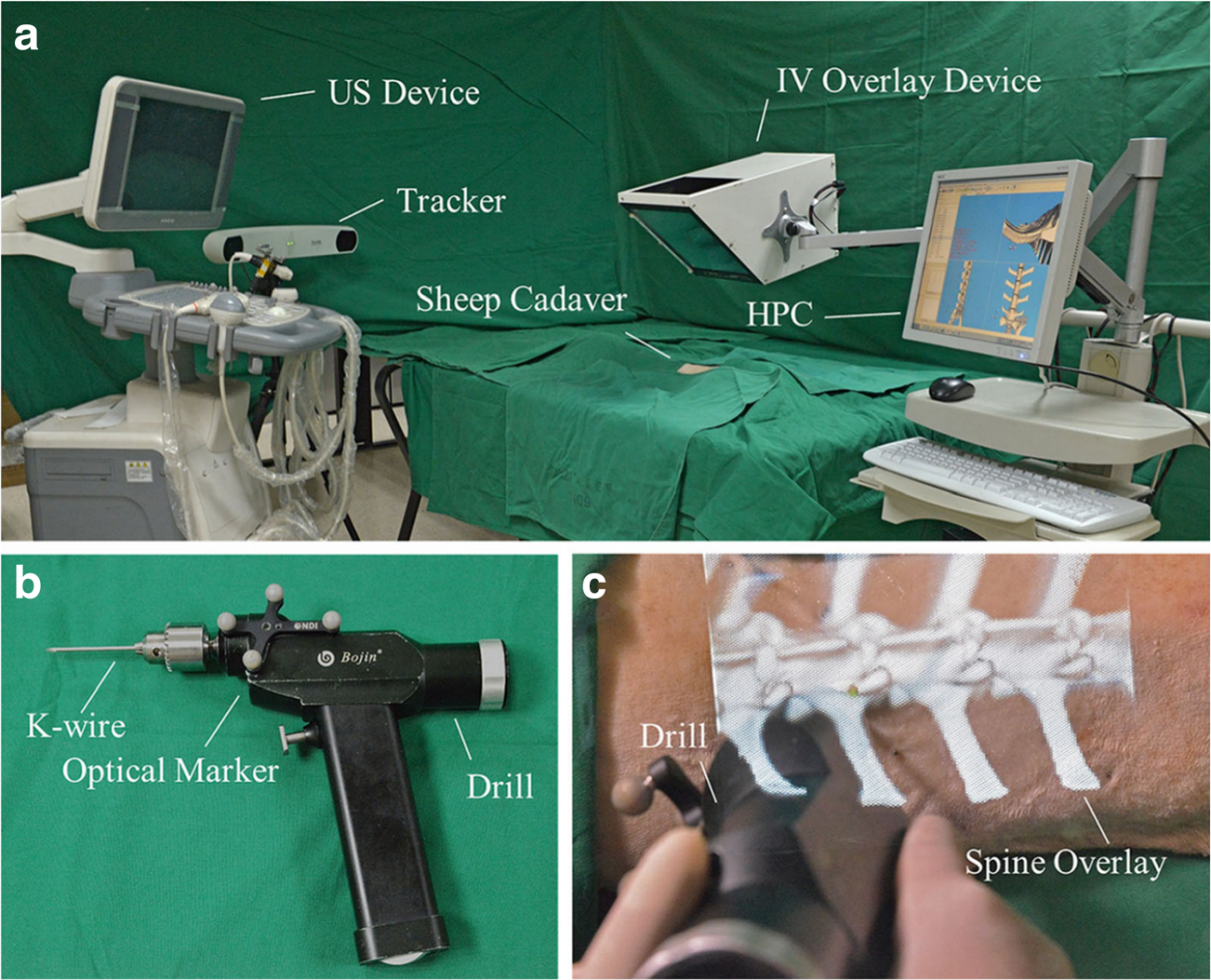
Example of a holographic visualization of augmented reality. In **a**, an overview of the setup. In **b**, the tracked drilling instrument. In **c**, the interface as seen by the surgeon. The figure is an unmodified reproduction from the work of Ma et al., published in the *International Journal of Computer Assisted Radiology and Surgery* [33]

Four main types of AR user interfaces were identified among the included studies. The two most common were monitor-based (Monitor-AR, 10 studies) and head-mounted displays (HMD-AR, 12 studies). Monitor-AR typically consisted of video cameras, imbedded in the C-arm, aimed at the surgical field and a separate monitor displaying the video feed with AR overlay (Fig. 2). HMD-AR, on the other hand, was worn on the surgeon’s head as goggles, and the AR view was overlaid directly in the surgeon’s field of view (Fig. 3). Microscope-based AR interfaces (Microscope-AR, 4 studies) projected pre-defined AR objects in the microscope view during microsurgery (Fig. 4). Projector-based AR interfaces (Projector-AR, 2 studies) provided holographic AR overlays on glass-screens situated between the surgical area and the surgeon (Fig. 5).

The heterogeneity among reported study outcomes did not allow meta-analytical comparisons between the AR interfaces. Accuracy measurements providing the distance between the planned trajectories and the final device position (i.e., technical accuracy) were provided in a majority of Monitor-, Projector-, and HMD-AR studies. However, there was no consensus on 1D, 2D, or 3D translational measurements or the anatomical plane when reporting 1D or 2D measurements. No study using Microscope-AR provided technical accuracy. Target registration error (TRE), describing the calculated reliability, or quality, of the patient registration in the navigation software, was reported instead. Since TRE is only one out of many parts contributing to the final technical accuracy, a direct comparison is not possible.

### Patient registration and tracking

Patient registration was performed in three principal ways. Ten studies used intraoperative cone beam computed tomography (CBCT), with simultaneous patient position registration, to align radiological imaging with the patient in the operating room (OR) [2, 8, 16–22, 38]. Eight studies used intraoperative computed tomography (CT) with simultaneous patient position registration [10–13, 32, 35, 36, 40]. The remaining studies used preoperative CT in some fashion, either coupled to manual alignment with hand gestures, fluoroscopy, or custom-made pointers to register bone surfaces in the OR [1, 23, 30, 33, 37, 42, 45, 46].

Overall, four different patient tracking technologies were used. All technologies relied on optical tracking, where optical or infrared cameras were used. Ten studies used optical markers attached to the patients’ skin, surrounding the surgical field [2, 8, 16–22, 38]. Nine studies used a dynamic reference frame usually attached to a spinous process or firmly attached to the patients’ skin [1, 10–13, 35–37, 40]. Seven studies relied on surface tracking combined with manual adjustments, where the camera system identified patients’ surface anatomy or exposed spinal anatomy [14, 23, 30, 31, 42, 45, 46]. One study relied on direct surface matching and tracking without the need for manual adjustments [44]. One study relied on ultrasonography to match internal bone surface anatomy to preoperative imaging [33].

### Accuracy and clinical outcomes

Seven studies compared AR to FH with or without fluoroscopy for pedicle screw placement [14, 18, 19, 22, 32, 38, 42]. The study with the highest available evidence grade was performed by Elmi-Terander et al., comparing a prospective cohort of 20 patients to 20 retrospectively enrolled patients where FH with or without fluoroscopy had been used [19]. The AR and FH groups were matched based on diagnosis and proportions of thoracic vs. lumbar screws. The study found a higher accuracy in the AR vs. the FH group (AR: 93.9% vs. FH: 89.6%, *p* < 0.05). The same authors used Gertzbein grading to compare AR-navigated and FH pedicle screw accuracies without fluoroscopic guidance in a cadaveric setup where AR had a superior accuracy (AR: 85% vs. FH: 64%, *p* < 0.05) [22]. Peh et al. performed a cadaveric study, using minimally invasive techniques, comparing AR to FH with fluoroscopy [38]. Overall, no significant difference was found between the groups (AR: 94% vs. FH: 88%, *p* = 0.50) but in secondary reported outcomes the trend was towards increased accuracy using AR. Although no power analysis was provided, the authors discussed that the study could have been underpowered. Comparing HMD-AR to FH with fluoroscopy, Urakov et al. observed fewer major breaches in the FH group (HMD-AR: 36.8% vs. FH: 0% major breaches) [42]. However, the study was small and carried no statistical analysis. Liu et al. compared HMD-AR to fluoroscopy-guided FH pedicle screws in a study on phantom models [32]. No significant difference was found (HMD-AR: 94% vs. FH: 100%, *p* = 0.106). Dennler et al. compared HMD-AR to FH in two groups of surgeons: novice or experienced spine surgeons [14]. They found no difference in accuracy between HMD-AR and FH in the experienced spine surgeon group, but superior accuracy for novice surgeons using HMD-AR (*p* = 0.044).

There were three studies comparing AR navigation to FH, with or without fluoroscopy, for spinal procedures other than pedicle screw placement. In a randomized controlled trial, Auloge et al. compared the accuracy of the pedicle cannulation phase in percutaneous vertebroplasty between two groups of 10 patients each, one using AR and the other FH with fluoroscopy [2]. They found no significant difference between the groups. In a similar setup, Wei et al. performed a randomized controlled trial on percutaneous kyphoplasty comparing HMD-AR to FH with fluoroscopy [46]. No accuracies were reported, but clinical outcomes differed significantly. The AR group had larger amounts of bone cement injected, increased postoperative vertebral height, and lower patient reported pain, 1 year postoperatively (*p* < 0.05 for all). Wanivenhaus et al. evaluated the benefit of AR for manual rod bending [45]. When comparing unassisted rod bending to using AR to display patient-adapted holographic rods to guide surgeons, time spent on bending and inserting the rod was significantly shorter with AR assistance (AR: 374 ± 79 vs. unassisted: 465 ± 121 s, *p* = 0.012). Rod length was also significantly more often correct with AR (AR: 15/18 vs. unassisted: 4/18, *p* < 0.001).

Only one study compared AR navigation to other modalities of navigated spine surgery. Müller et al. used an HMD-AR device to place pedicle screws [37]. The control group consisted of patients treated using a widely available pose-tracking system (PTS) based on infrared cameras. There were no significant differences in translational errors (AR: 3.4 ± 1.6 mm vs. PTS: 3.2 ± 2.0 mm, *p* = 0.85) or angular errors (AR: 4.3 ± 2.3° vs. PTS: 3.5 ± 1.4°, *p* = 0.30).

### Workflow

Three studies reported shorter and one longer operating times when using AR compared to FH with or without fluoroscopy [2, 32, 45, 46]. Ten studies reported favorable impact on surgical time but lacked controls, or reported qualitatively positive workflow results for AR navigation [11, 13, 20, 21, 23, 30, 36, 38, 42].

### Radiation dose

All studies reporting on radiation doses used either intraoperative CT or intraoperative CBCT for patient registration (Table 2). No study relying on preoperative CT or intraoperative fluoroscopy for patient registration reported radiation doses.

**Table 2:** Studies reporting radiation doses

Authors	Year	Patient registration type	Surgical procedure	Radiation including postoperative verification	Radiation dose to patient	Radiation dose to staff
Auloge et al. [2]	2019	CBCT Intraop	Vertebroplasty, 1 level	No	DAP: 182.6 ± 106.7 mGy.71^2^	n/a
Carl et al. [10]*	2019	CT Intraop	Variable, 1–2 vertebral levels*	No	ED, mean dose: Cervical: 0.52 mSv Thoracic: 6.14 mSv Lumbar: 2.99 mSv	n/a
Carl et al. [11]*	2019	CT Intraop	Intradural spinal lesions, 1–4 levels*	No	ED, mean dose: Cervical: 0.22 ± 0.16 mSv Thoracic: 1.68 ± 0.61 mSv	n/a
Carl et al. [13]*	2019	CT Intraop	Extra- and intradural spinal lesions, 5–13 levels*	No	ED, range (min–max): Cervical: 0.35radur mSv Thoracic: 2.16radur mSv Lumbar: 3.5516rad mSv	n/a
Carl et al. [12]*	2020	CT Intraop	Extra- and intradural lesions, degenerative, infections, and deformities*	No	ED, mean dose: Cervical: 0.29 ± 0.17 mSv Thoracic: 3.40 ± 2.38 mSv Lumbar 3.05 ± 0.89 mSv	n/a
Edstrom et al. [17]	2020	CBCT Intraop	Mainly scoliosis, 2–12 levels	Yes	ED, average: 15.8 ± 1.8 mSv	Staff dose, average: 0.21 ± 0.06 μSv
Peh et al. [38]	2020	CBCT Intraop	Cadaveric pedicle screw placement	Yes	n/a	“The performing surgeon was not exposed to radiationt

*CBCT* cone beam computed tomography, *CT* computed tomography, *DAP* dose-area product, *ED* effective dose

*A large share of patients has been re-used in these studies

One study compared AR navigation with CBCT patient registration to FH with fluoroscopy, and found that AR navigation resulted in significantly lower dose-area product (AR: 182.6 ± 106.7 mGy cm^2^ vs. FH: 367.8 ± 184.7 mGy cm^2^, *p* = 0.025) and fluoroscopy time (AR: 5.2 ± 2.6 s vs. FH: 10.4 ± 4.1 s, *p* = 0.005) [2].

Five studies using either intraoperative CT or CBCT for patient registration reported effective dose, with means between 0.22 ± 0.16 mSv (cervical) and 15.8 ± 1.8 mSv (thoracolumbar) [10–13, 17]. Four of those studies were based partially, or in whole, on the same patient cohort, however [10–13]. Two studies using intraoperative CBCT for patient registration highlighted that staff radiation was null or negligible, due to being fully shielded while using radiation or because none was used intraoperatively [17, 38].

### Cost-benefit

No included study provided cost-benefit analyses of using AR navigation or discussed costs and benefits in quantitative terms.

## Discussion

The use of AR navigation in spine surgery represents a meaningful improvement over existing CAS technologies concerning workflow and ease-of-use and is favorable to FH surgery in terms of accuracy and radiation exposure. AR systems have demonstrated a high accuracy compared to FH surgery in several clinical studies. Notably, all studies involving pedicle screw placement accuracy that reported favorable results for AR relied on Monitor-AR. HMD-AR was either comparable or inferior to FH surgery for pedicle screw placement. This may be explained by the fact that HMD systems are comparatively newer and have one additional tracked object (the HMD itself), thereby increasing the complexity and potential for errors. Another explanation could be that all Monitor-AR included in this systematic review relies on intraoperative CBCT and skin markers for patient registration and tracking, while most HMD-AR relies on manual registration and direct patient surface tracking. When using manual registration and direct surface tracking, the AR image is manually adjusted to match the reality thereby inducing a potential registration error. Intraoperative imaging in combination with optical markers or a DRF, however, allows for an accurate automatic co-registration [7, 10]. Nonetheless, an isolated comparison between Monitor-AR and HMD-AR on the one hand, and optical markers and surface tracking on the other hand, cannot be performed on the currently available data.

An ideal navigation system for spine surgery should provide a clear interface highlighting only what is important and an unobtrusive patient tracking with high fault-tolerance. Proponents of HMD-AR may argue that it will represent the optimal user experience once the technology is matured, provided it reaches an acceptable accuracy. If the HMD device is unobtrusive and lightweight and offers a wide field of view, having the AR overlay directly in the surgeon’s view could be an advantage. However, HMD may increase the risk for inattentional blindness compared to monitors [15]. Nonetheless, as long as accuracy, bulkiness, or other practical factors inhibit this end-goal, Monitor-AR may be the better alternative. The advantage of a monitor is that the surgeon can perform other tasks in the OR that do not require navigation, without being disturbed by a head piece. The results of this systematic review are not conclusive regarding the best interface.

In this systematic review, the impact of AR navigation on radiation exposure for both patients and staff were favorable compared to FH. Given that AR principally concerns presentation of imaging data, the radiation exposure is expected to be comparable to other CAS solutions. However, improved workflow may reduce staff exposure, while increased accuracy may reduce the need for additional imaging.

Notably, no study provided any cost-benefit analyses of using AR navigation. However, the financial benefits of navigation were presented in a recent review indicating that using navigation results in “buying-back” the investment in the long term [26]. There is no reason for AR systems to deviate from this pattern. In the future, it will be paramount to include financial evaluations of each system studied.

### Future perspectives

An ideal spinal navigation system should provide a time-efficient setup and registration, be easy to use, and allow visualization of the anatomy without distracting the surgeon or obscuring the surgical field [25, 26]. The system must be accurate and preferably offer the possibility to confirm the results. AR navigation offers a solution to increase the ease-of-use while allowing an unobtrusive visualization of the anatomy. Using optical markers or surface recognition for patient tracking simplifies the setup and registration process.

The obtained technical accuracy by AR is already relatively high. The challenge is to achieve maximal accuracy also in complex cases. By including AR tracking of the surgical tools, a more direct feedback could be achieved [8]. Moreover, surgical accuracy could be markedly improved by replacing the human hand with a robotic arm. Initial studies on AR navigation combined with robotics demonstrate a significantly higher accuracy than AR without the robot [4, 5]. Automatization of parts of the process using AI or machine learning could both improve workflow and simplify robotic integration [6].

To further improve surgical results, AR navigation could be combined with sensing technologies such as impedance probes or optical probes relying on diffuse reflectance spectroscopy (DRS) [9, 39]. These, and similar, technologies could provide direct feedback on the tissue type where the tip of the surgical tool or screws are located and possibly be integrated in an automated workflow.

Arguably, it is only a matter of time until technological achievements transferred to spine surgery will have the upper hand in terms of accuracy in identifying static anatomical landmarks and eventually also dynamic or moving surgical targets. The boundaries between what can and what cannot be done with computer assistance, robotics, and AI in surgery will primarily be defined by medico-legal concerns rather than technological. However, for a foreseeable time, the surgical handicraft needed for decompression and microneurosurgical handling of intrathecal nervous system tissues will be reserved for the surgeon. Nonetheless, AR visualization may provide the surgeon with valuable assistance in the performance of these delicate maneuvers.

### Limitations

AR in spine surgery is a relatively new concept reflected in the limited number of publications. Most included studies were non-controlled or non-randomized, which could introduce a potential bias in the study outcomes and conclusions. Only one RCT was identified in our systematic review. Only one study was publicly registered before beginning the trial and no additional studies could be found by the authors of this review on clinicaltrials.gov, meaning the ability to objectively evaluate publication bias is currently lacking.

## Conclusions

AR provides a meaningful addition to FH surgery and traditional navigation methods for spine surgery. By enhancing the surgical field with radiological guidance information, the surgeon’s attention is kept on the surgical field. In this systematic review, superior workflow and non-inferior accuracy were the main findings when comparing AR to FH or conventional navigation techniques. A limited number of studies indicated decreased radiation dose for both patients and staff.

Future developmental efforts should be focused on further improving the AR system setups regarding workflow optimization and choice of the method to present virtual information. Future studies on the impact on clinical outcomes such as patient mortality, morbidity, and complications are required.
